# Impact of the statutory concessionary travel scheme on bus travel among older people: a natural experiment from England

**DOI:** 10.1017/S0144686X19000692

**Published:** 2019-07-18

**Authors:** Elise Whitley, Peter Craig, Frank Popham

**Affiliations:** MRC/CSO Social and Public Health Sciences Unit, University of Glasgow, Glasgow, UK

**Keywords:** ageing, concessionary travel, public transport, bus travel, natural experiment

## Abstract

In the context of worldwide ageing, increasing numbers of older people are lonely, isolated and excluded, with serious implications for health, and cognitive and physical functioning. Access to good public transport can improve mobility and social participation among older adults, and policies that improve access and promote use, such as concessionary travel schemes, are potentially important in promoting healthy and successful ageing. Concessionary travel schemes for older people are in place in many countries but are under threat following the global financial crisis. Evidence regarding their success in encouraging activity and social participation is generally positive but based largely on qualitative or observational associations and, in particular, is often limited by the lack of appropriate comparison groups. We use changes in the English statutory scheme, in particular the rising eligibility age from 2010 onwards, as a natural experiment to explore its impact on older people’s travel. A difference-in-difference-in-difference analysis of National Travel Surveys (2002–2016) compares three age groups differentially affected by eligibility criteria: 50–59 years (consistently ineligible), 60–64 years (decreasing eligibility from 2010) and 65–74 years (consistently eligible). Compared with 50–59-year-olds, bus travel by 60–74-year-olds increased year-on-year from 2002 to 2010 then fell following rises in eligibility age (annual change in weekly bus travel: −2.9 per cent (−4.1%, −1.7%) in 60–74- *versus* 50–59-year-olds). Results were consistent across gender, occupation and rurality. Our results indicate that access to, specifically, free travel increases bus use and access to services among older people, potentially improving mobility, social participation and health. However, the rising eligibility age in England has led to a reduction in bus travel in older people, including those not directly affected by the change, demonstrating that the positive impact of the concession goes beyond those who are eligible. Future work should explore the cost–benefit trade-off of this and similar schemes worldwide.

## Introduction

With populations ageing worldwide, there is a recognised problem of loneliness, social isolation and exclusion among older people (Age UK, 2011). In addition to obvious impacts on wellbeing, there are serious health implications, with loneliness prospectively associated with depression, dementia progression, impaired cognition and functioning, and outcomes such as hypertension, heart disease, and stroke (Ong *et al*., 2016). Policies and initiatives supporting social engagement and activity among older people are therefore important for promoting healthy and successful ageing. The importance of environment in determining health outcomes is increasingly well recognised, with the World Health Organization (WHO) International Classification of Functioning, Disability, and Health (WHO, 2013) specifically including an ‘environmental factors’ component, covering aspects such as technology, natural environment, and services, systems and policies, and an environmental factor of particular relevance to ageing populations is access to public transport. Private car is a popular transport mode but is known to decrease with age as result of increasing physical and mental limitations as well as major life events such as retirement or bereavement (Shrestha *et al.*, 2017) (*e.g.* in England approximately 80% of individuals hold a full driving licence compared with around 60% of those aged 70+; https://www.gov.uk/government/statistical-data-sets/nts02-driving-licence-holders) and driving cessation has been shown to be associated with loss of independence and social identity (Pachana *et al.*, 2017), decreased social engagement (Curl *et al.*, 2014) and increased health problems, most markedly depression (Chihuri *et al.*, 2016). Patterns of transport use vary across countries but reviews of international evidence suggest that access to public transport is consistently associated with increased mobility and social participation among older adults (Levasseur *et al.*, 2015; Vaughan *et al.*, 2016), and the WHO includes affordable, reliable and accessible public transport in their Checklist of Essential Features of Age-friendly Cities (WHO, 2007). Policies that improve access and encourage greater use of public transport therefore have great potential to improve the health and wellbeing of older people. One such policy is the introduction of concessionary travel schemes offering free or reduced-cost public transport travel to older people, which are in place in many countries (GOAL Consortium, 2012; Organisation for Economic Co-operation and Development (OECD), 2015). The costs, benefits and terms of these schemes vary from country to country but, in general, associations with mobility and social participation are positive. Free or reduced-cost access to public transport removes financial constraints to travel and has been shown to increase social engagement among older people by enabling them to visit friends and family, volunteer, or attend events and activities (Reinhard *et al.*, 2018). In addition, results from qualitative studies (Hirst and Harrop, 2011; Andrews, 2012; Andrews *et al.*, 2012; Green *et al*., 2014) suggest that bus travel is often regarded as a social experience in itself. More broadly, bus pass ownership has been associated with increased physical activity (Coronini-Cronberg *et al.*, 2012; Webb *et al.*, 2016) and reduced rates of obesity (Webb *et al.*, 2012), and increased public transport use as a result of concessionary schemes is associated with reductions in loneliness and depressive symptoms (Reinhard *et al.*, 2018).

While studies exploring the impacts of concessionary travel schemes have generally been positive, a recent review (Mackett, 2014*a*) highlights their limitations, including restriction to specific groups of people or local areas, and the tendency for concessionary schemes to coincide with retirement when individuals change their lifestyle, making it difficult to attribute changes in travel patterns specifically to the availability of concessionary travel (Siren and Haustein, 2016). In addition, the schemes are argued to be costly, *e.g.* the current English scheme’s estimated cost is over £1 billion per annum (Butcher, 2015), and it is therefore important to understand whether there are particular groups that benefit more than others. For example, bus travel is more common in women, those in urban areas and those without access to a car (Mackett, 2014*b*), and these groups may benefit more, as may people on lower incomes who have less disposable income to use on non-essential travel. However, the greatest challenge in studies of this type is the identification of a suitable comparison group as a robust evaluation ‘require[s] a large survey of those with passes and a similar population without … but this cannot be done because [these schemes represent] a universal benefit’ (Mackett, 2014*a*). Additionally, concessionary transport ‘interventions’ are something of a moving target, with differences in schemes’ eligibility criteria and benefits occurring across countries and over time. However, when accounted for in the study design, these differences can provide the comparison groups required for robust evaluation.

In this paper, we exploit changes in the English concessionary travel scheme as a natural experiment to understand its impact on public transport use by older people. In particular, we focus on the introduction of a statutory scheme that replaced previous local discretionary schemes in order to set ‘a national minimum standard for local authority concessionary fares schemes for elderly people’ (Butcher, 2015: 4). The stated objectives of this scheme were to increase older people’s public transport use and access to services such as shops and health care, reduce social isolation/exclusion and promote wellbeing in older age (Mackett, 2014*a*). The scheme primarily covers travel on local bus services outside peak times although some local authorities offer greater benefits, *e.g.* the London Freedom Pass allows free travel on almost all public transport including trains, underground and trams, often including peak times (https://www.londoncouncils.gov.uk/services/freedom-pass/using-pass). The scheme is popular (*e.g.* in 2013 uptake in those eligible on the basis of age was around 80% and a third of all bus trips in England were taken by people travelling free through age and/or disability), with greater uptake among women and those living in more urban areas, particularly London (Mackett, 2013). The statutory scheme was introduced in April 2001, offering halfprice local bus travel to pass holders aged 60+ (women) or 65+ (men), with the eligibility age equalised to 60+ in both sexes in April 2003 (Butcher, 2015). The scheme was revised to allow free local bus travel in April 2006 and additionally extended in April 2008 to provide free travel on local buses throughout England. More recent legislation has brought the eligibility age in line with the female state pension age, rising from 60 in 2010 to (projected) 66 in 2020. We use a difference-in-difference-in-difference design and data from the National Travel Surveys (NTS) (Department for Transport, 2017) to explore variation in travel patterns over time in groups differentially impacted by changes in the scheme. In particular, we consider the changing benefits offered by the scheme and exploit the raising of the eligibility age from 2010 onwards, representing a graded withdrawal of the concession from later cohorts of 60–64-year-olds (Craig *et al.*, 2018). Similar approaches have been used previously to explore specific changes to the concessionary schemes (Kelly, 2011; Houston and Tilleyed, 2016) but there have been issues of non-comparability of intervention and control groups. We consider year-on-year changes in consistent age bands defining three populations based on eligibility for concessionary travel: (a) those consistently ineligible, (b) those consistently eligible and (c) those with decreasing eligibility between 2010 and 2016. Our research aims were: (a) to understand the impact of the statutory concessionary travel scheme on public transport use by older people and (b) how this varies by gender, urban/rural location, occupation and car ownership.

## Methods

Details of the NTS are provided in annual technical reports (Lepanjuuri *et al.*, 2017). The NTS is a series of household surveys performed annually since 1988. Early surveys were based on ~5,000 addresses per year, rising to ~15,000 in 2002. Until recently, households were sampled from all of Great Britain but in 2013 coverage was restricted to England. All household members (up to a maximum of ten) are invited to take part, with annual response rates around 60 per cent. Participants are interviewed and most also complete a travel diary recording details of every journey made during a seven-day period. The current analyses are based on data from English households surveyed between 2002 and 2016, when comparable questions were asked each year. All analyses use NTS weights to ensure that they are generalisable to the English population (Lepanjuuri *et al.*, 2017).

We explored the impact of two ‘interventions’ (Table 1). The first, considering benefit levels, compares 2002–2005, when travel was half-price, with 2006–2009, when travel was free. The second, concerning eligibility, compares 2006–2009, when all 60–74-year-olds were eligible, *versus* 2010–2016, when the eligibility age for free travel rose from 60 to 63, resulting in decreasing eligibility in this age group. In each case the control group was 50–59-year-olds, who were not eligible for concessionary travel on the basis of age, and the intervention groups were those aged 60–64 and 65–74. We used a difference-in-difference-in-difference approach to compare yearly changes before and after the interventions and between the intervention and control groups (*i.e.* differences across year, time period and age group), allowing greater understanding of the incremental impact of changes to the scheme. Specifically, we fitted regression models for outcomes in terms of survey year (continuous), intervention period (pre- or post-intervention) and age group (50–59, 60–64, 65–74):

$$\begin{array}{l} Y_{ijk}=\alpha_{1}{\text{Survey}\;\text{year}}_{i}+\alpha_{2}\text{Pre-}/{\text{Post-intervention}}_{\text{j}}+\alpha_{3}{\text{Age}\;\text{group}}_{\text{k}}\\ \;+\beta_{1}\left({\text{Survey}\;\text{year}}_{i}\times \text{Pre-}/{\text{Post-intervention}}_{\text{j}}\right)\\ \;+\beta_{2}\left({\text{Survey}\;\text{year}}_{i}\times {\text{Age}\;\text{group}}_{k}\right)\\ \;+\beta_{3}\left(\text{Pre-}/{\text{Post-intervention}}_{\text{j}}\times {\text{Age}\;\text{group}}_{\text{k}}\right)\\ \;+\gamma \left({\text{Survey}\;\text{year}}_{i}\times \text{Pre-}/{\text{Post-intervention}}_{\text{j}}\times {\text{Age}\;\text{group}}_{\text{k}}\right)+{\text{ε}}_{\text{ijk}} \end{array}$$

with the impact of the intervention estimated by $\hat{\gamma }$, the differential year-on-year change in outcome after the intervention in the intervention groups. Propensity score weights based on sex, car access, rurality and occupation were used to account for changes in group composition arising from the use of repeated cross-sectional data (Stuart *et al.*, 2014).

Interview data were used to explore the proportion of respondents reporting that they owned a (‘OAP’– Old Age Pensioner) bus pass and the proportion travelling by bus at least weekly. The purpose of any public transport travel was investigated using seven-day diary data, considering the proportion of respondents recording any journeys made by bus or, in London, underground (reflecting the benefits offered by the London Freedom Pass), for (a) any purpose and (b) shopping or access to services (including medical consultations/treatment, banks, libraries, churches, hairdressers, launderettes, dry-cleaners, betting shops, solicitors, estate agents). All analyses were performed for all respondents combined and, separately, by sex, car access (driver with access to car *versus* non-driver/driver with no access), rurality (based on 2011 census classification: urban *versus* rural) and occupation (based on National Statistics classification: managerial/professional/intermediate *versus* routine/manual/never worked/unclassified). Results from sensitivity analyses considering 65–69- rather than 65–74-year-olds or excluding London underground travel were very similar to those presented here.

## Results

Interview data were available for 34,987 respondents aged 50–59, 16,706 aged 60–64 and 26,952 aged 65–74 (Table 2). The three age groups were broadly similar in terms of sex and rurality. However, older respondents were increasingly more likely to have/have had a manual occupation and no access to a car. Considering all survey years combined, none of those aged 50–59 reported having a bus pass compared with 51 per cent of those aged 60–64 and 74 per cent of those aged 65–74. Similarly, 19, 27 and 35 per cent of the 50–59-, 60–64- and 65–74-year-olds, respectively, reported travelling by bus at least weekly. Data from travel diaries were available for 30,369, 14,828 and 24,138 respondents aged 50–59, 60–64 and 65–74, respectively. The proportion of respondents recording at least one journey by bus or London underground was similar to the proportion reporting weekly bus travel.

**Figure 1:**
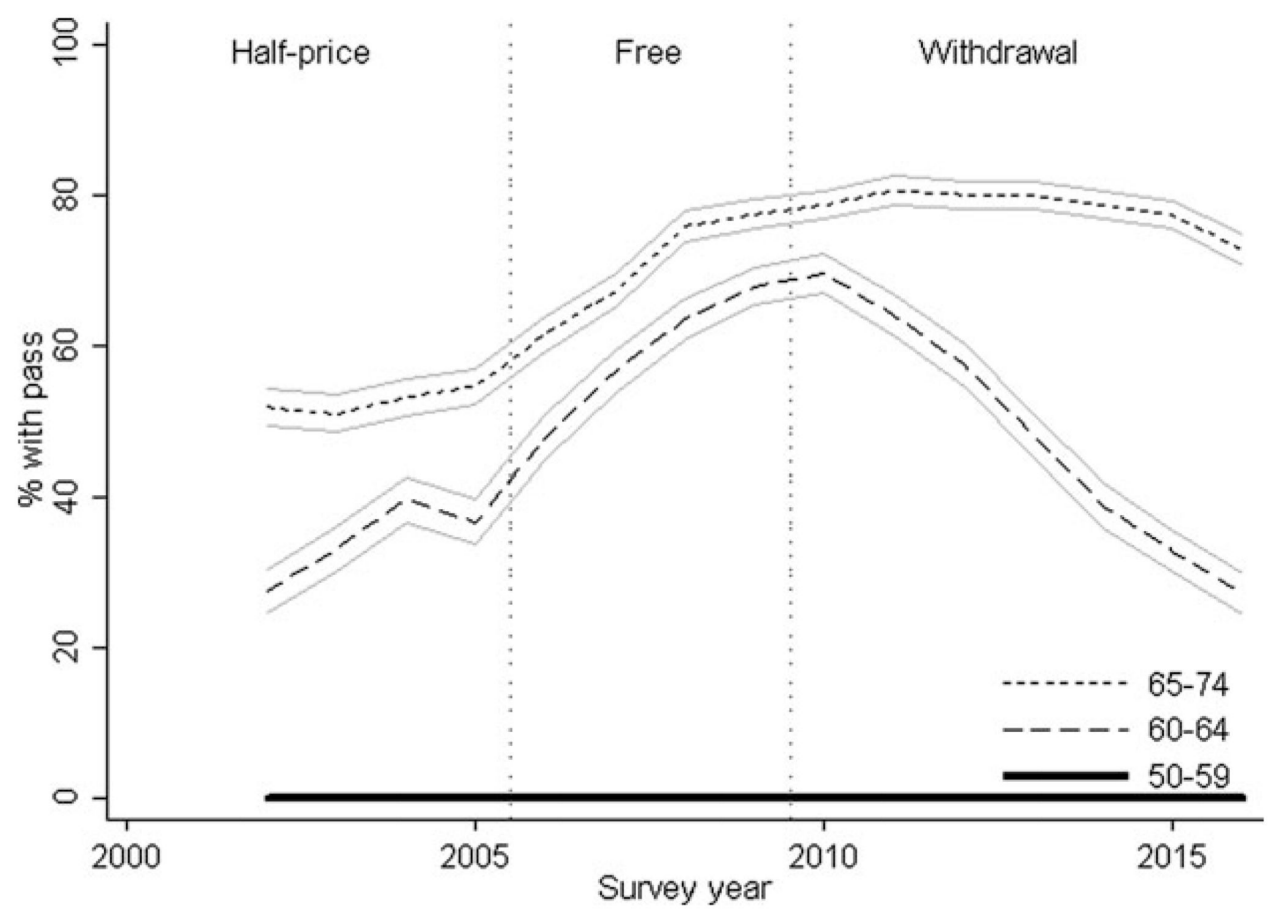
Self-reported bus pass ownership according to survey year and age group. *Note*: Ninety-five per cent confidence intervals are shown.

Figure 1 presents proportions of self-reported bus pass ownership and corresponding 95 per cent confidence intervals (CI) (*see* associated statistics in Table S1 in the online supplementary material). As expected, there was no bus pass ownership among 50–59-year-olds. Ownership among 65–74-year-olds was consistently higher than for 60–64-year-olds and increased steadily in both older groups during the period of half-price travel, with more marked increases when travel became free (pre- *versus* post-intervention yearly change in those aged 60–64 and 65–74 *versus* 50–59: 3.4% (95% CI = 1.7, 5.0) and 4.5% (95% CI = 2.9, 6.1), respectively; *p* < 0.001). From 2010 onwards, ownership among 65–74-year-olds plateaued before falling slightly while the rate among 60–64-year-olds fell sharply from around 60 per cent in 2010 to less than 30 per cent in 2016 (yearly change: −14.1% (95% CI = −15.1, −13.0) and −6.6% (95% CI = −7.6, −5.5) in those aged 60–64 and 65–74 *versus* 50–59, respectively; *p* < 0.001). Similar patterns were observed for men and women, urban and rural dwellers, those with manual and non-manual occupations, and with and without access to a car (*see* Figure S1 in the online supplementary material).

**Figure 2:**
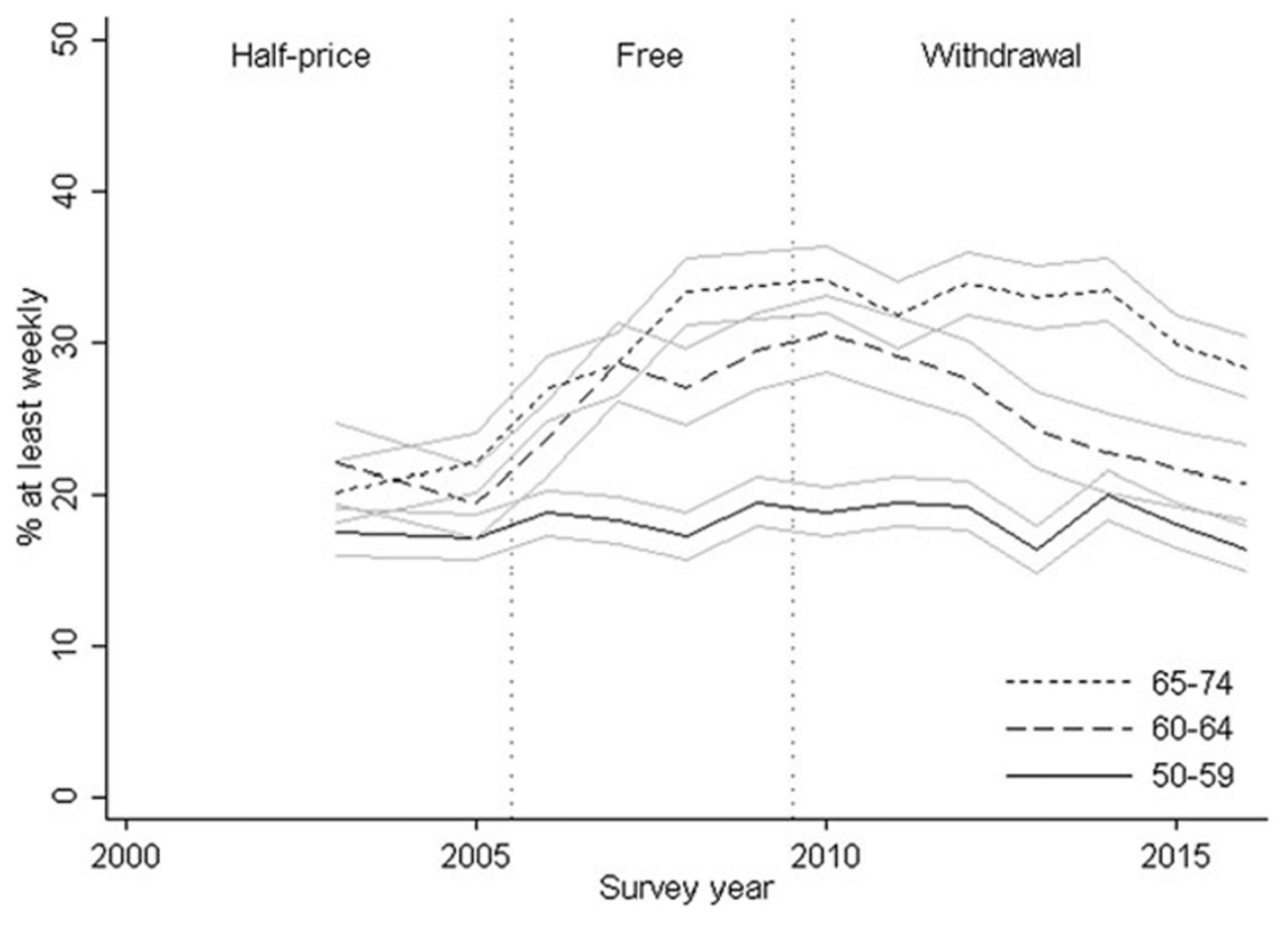
Self-reported weekly bus use according to survey year and age group. *Note*: Ninety-five per cent confidence intervals are shown.

**Figure 3:**
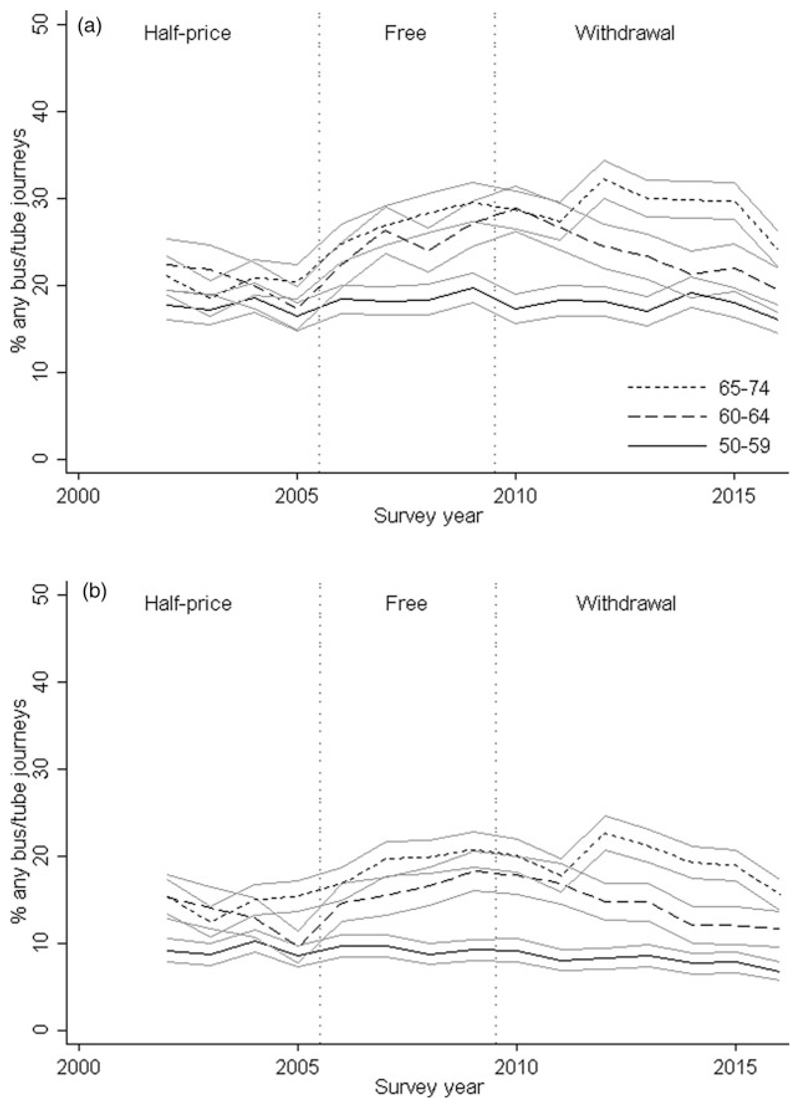
At least one journey recorded in weekly diary by bus or London underground according to survey year and age groups; journeys for (a) all purposes and (b) shopping or access to services. *Note*: Ninety-five per cent confidence intervals are shown.

The proportion of respondents travelling by bus at least weekly is presented in Figure 2 (*see* Table S2 in the online supplementary material). Less than 20 per cent of 50–59 year olds reported using buses weekly and this was broadly consistent over time. Weekly bus travel rates were higher in 60–64 and 65–74 year olds, increasing in both groups when travel became free (yearly change: 2.6% (95% CI = 0.0, 4.9) and 1.2% (95% CI = −0.1, 3.5) in those aged 60–64 and 65–74 *versus* 50–59, respectively; *p* = 0.09). Weekly bus travel decreased in both groups from 2010 onwards (yearly change: −2.9% (95% CI = −4.1, −1.7) in both those aged 60–64 and 65–74 *versus* 50–59; *p* < 0.001). Women, respondents with manual occupations or living in urban areas were more likely to report weekly bus use but patterns of use over time were similar to those in Figure 3 (*see* Figure S2 in the online supplementary material). The same was true of respondents with access to a car. However, weekly bus travel rates were markedly higher (50–60%) among respondents with no car access and remained broadly similar across the three age groups, with a slight increase in the two oldest groups between 2005 and 2010 followed by a levelling off or slight decrease in both groups thereafter.

Figure 3 (*see* Tables S3a and S3b in the online supplementary material) presents the proportion of respondents with at least one journey by bus/London underground recorded in seven-day diaries for (a) any reason and (b) shopping/access to services. Results for all journeys were very similar to those for self-reported weekly bus travel. Approximately 30 per cent of journeys made by 50–59-year-olds were for shopping/access to services compared with around 50 per cent in 65– 74-year-olds. Patterns over time in the three age groups were very similar to those for all journeys combined. Bus/underground travel was broadly consistent in 50–59-year-olds, with a slight fall from 2010 onwards, but increased in older groups following the introduction of free travel (yearly change: 3.2% (95% CI = 1.7, 4.7) and 1.0% (95% CI = −0.5, 2.5) in those aged 60–64 and 65–74 *versus* 50–59, respectively; *p* < 0.001). Rates decreased in the older groups from 2010 onwards, most markedly in those aged 60–64-year-olds (yearly change: −2.2% (95% CI = −3.3, −1.2) and −1.7% (95% CI = −2.7, −0.6) in those aged 60–64 and 65–74 *versus* 50–59, respectively; *p* < 0.001).

## Discussion

International evidence suggests that access to public transport is associated with increased social participation and mobility among older adults (Levasseur *et al.*, 2015; Vaughan *et al.*, 2016) and the provision of affordable, reliable and accessible public transport is included in the WHO Checklist of Essential Features of Age-friendly Cities (WHO, 2007). Concessionary travel schemes exist in many countries (GOAL Consortium, 2012; OECD, 2015) and bus pass ownership and associated public transport travel have been associated with increased physical activity and lower rates of obesity, loneliness and depressive symptoms (Coronini-Cronberg *et al.*, 2012; Reinhard *et al.*, 2018; Webb *et al.*, 2012, 2016). A number of previous studies (Halcrow Group Limited, 2009; Passenger Focus, 2009; Rye and Mykura, 2009; Hirst and Harrop, 2011; Kelly, 2011; Andrews, 2012; Andrews *et al.*, 2012; Jones *et al.*, 2013; Shaw and Hewitt, 2013; Green *et al.*, 2014; Shaw and Hewitt, 2014; Houston and Tilleyed, 2016) have considered the success of concessionary schemes in increasing pass uptake and public transport use among older people but have been limited by the lack of appropriate comparison groups coupled with changes in the schemes over time (Mackett, 2014*a*). We have exploited these changes using a difference-in-difference-in-difference design. In contrast to consistent rates among 50–59-year-olds, bus pass ownership and travel increased among 60–74-year-olds following the introduction of free travel. The gradual withdrawal of concessionary travel in 60–64-year-olds resulted in decreasing ownership and travel in this group, and travel also decreased among 65–74-year-olds following this change, with sensitivity analyses confirming that these results were not driven by the London scheme or by the specific choice of age group. Results were consistent across gender, occupation and rurality; only those without access to a car were unaffected by changes to the scheme.

The difference-in-difference-in-difference approach takes into account underlying differences in characteristics between the groups that remain the same at all times, providing more robust estimates of effect than designs that only control for a limited number of these differences. In addition, the approach controls for temporal changes that affect all study participants, *e.g.* changes to bus services, extreme weather events, recession or changes in data collection. However, it is possible that changes in specific age groups, *e.g.* increasing car use among older people over time, could account for some of our results, although separate analyses (not shown) indicate that changes in car use is not a factor. There are also some limitations in the data-set used. Results are based on existing survey data and variables did not always provide the exact information we require; in particular, we lack direct information on disposable income, social isolation, wellbeing and health. Furthermore, data on the purpose of journeys was only available at the aggregate level (shopping or access to services) and it was not possible to look at individual destinations, some of which are likely to be more essential than others. In addition, analyses are based on repeated cross-sectional data rather than following a single cohort over time, although we used propensity score weights to account for potential changes in the balance of key characteristics between groups over time (Stuart *et al.*, 2014). In addition, the use of NTS weights means that our results are representative of the English population and therefore characterise the impact of concessionary travel at a national level, although we were not able to make comparisons over the same time period with schemes in Wales and Scotland.

Driving cessation is increasingly common among older people (Shrestha *et al.*, 2017) with negative implications for independence, social identity (Pachana *et al.*, 2017), social engagement (Curl *et al.*, 2014), and physical and mental health (Chihuri *et al.*, 2016). However, public transport offers an alternative means of travel for those who no longer drive and our results suggest that access to free travel increases bus pass ownership and bus travel among older people. Taken in combination with existing evidence of positive health associations with bus pass ownership and public transport use (Coronini-Cronberg *et al.*, 2012; Webb *et al.*, 2012, 2016; Reinhard *et al.*, 2018), these results provide strong support for the success of the free travel scheme in improving public transport use, access to services, social engagement, wellbeing and health among older people. However, results for the period in which the concession offered half-price travel were less striking. Bus pass ownership increased during this time, although less markedly than the period in which the concession offered free travel, but there was little evidence of an associated impact on bus travel, which increased markedly only when travel was free. Concessionary schemes are in place in many countries (GOAL Consortium, 2012; OECD, 2015) but the terms and benefits vary and, in particular, not all offer free travel. Our results demonstrate a specific impact of free travel on bus use in England, where private car travel is popular. However, the impact of other schemes on social participation and mobility among older people may vary according to the benefits offered, the relative cost of public and private transport, and societal conventions regarding their use. Further international comparisons are therefore required to better understand the benefits of different country-specific schemes.

The impact of the recent raising of age of eligibility for free travel in England has not been widely explored. Our results show a clear decrease in bus pass ownership among those directly affected by the change, with a corresponding decrease in bus travel indicating that the concession withdrawal has had a negative impact. Moreover, while bus pass ownership in those still eligible has plateaued, it is of note that bus travel among 65–74-year-olds has also decreased since the policy change, suggesting that there is a spill-over effect. This could be a result of reduced popularity of a less universal scheme and, in the most recent years, there has been a small reduction in bus pass ownership in the oldest group. Alternatively, couples or social groups may be responding to lack of access to free travel in one or more of their members by opting, as a whole, not to travel or to use private transport instead. These results suggest that the impact of concessionary schemes may extend beyond those who are directly eligible. It is therefore important to recognise that policies that reduce eligibility may have an impact beyond those directly affected, with serious consequences for the social engagement, wellbeing and health of many older people, and economic evaluations of international concessionary schemes should recognise this potentially broader impact.

The use of public transport varies across different groups (Mackett, 2014*b*). Our results showed expected patterns of greater bus use by women and individuals living in urban areas, with no access to a car, and with manual occupations and therefore potentially less disposable income to access more expensive modes such as train travel or taxis. However, changes to the scheme generally had a very similar impact across groups, suggesting that concessionary travel encourages additional trips regardless of sex, occupation, income or rurality. Previous work has shown that car ownership is a key factor in determining inequalities in travel (Mackett, 2014*b*). However, our results suggest that concessionary schemes may also increase bus use among those with car access. Shifting from private to public transport has many advantages, *e.g.* increasing social engagement and physical activity (Coronini-Cronberg *et al.*, 2012; Webb *et al.*, 2016; Reinhard *et al.*, 2018), and, more broadly, reducing congestion, emissions and road traffic accidents. The group in which the concessionary scheme had least impact was those with no access to a car. Unsurprisingly, this group had the highest rates of bus travel overall and there was little evidence that raising the age of eligibility had any impact, suggesting that public transport was a necessity rather than a choice. However, it is worth noting that, following the introduction of free travel, bus use among older people in this group also increased, suggesting an increase in the number of non-essential journeys, which are more likely to be made for social purposes.

## Conclusion

Previous international evidence suggests that access to public transport is associated with increased social participation and mobility among older people. Our empirical results suggest that schemes providing, specifically, free travel are effective in promoting public transport use, increasing access to services and, potentially, improving social engagement, wellbeing and health among older people. In addition, the positive impact is evident across sex, occupation and rurality, and may encourage additional bus travel among older car owners. However, the recent rise in eligibility age in England has impacted negatively on bus travel beyond those directly affected. Given international government priorities for promoting successful ageing and reducing loneliness, further work is needed to understand the costs and benefits of different country-specific schemes.

## Supplementary Material

**Supplementary material.** The supplementary material for this article can be found at https://doi.org/10.1017/S0144686X19000692.

Supplementary

## Figures and Tables

**Table 1:** Statutory concessionary travel scheme in England and numbers in analytical sample based on 50–74-year-olds living in England and surveyed between 2002 and 2016

			Control group		Intervention groups			
				
			Age 50–59 with data from:		Age 60–64 with data from:		Age 65–74 with data from:	
					
Survey year	Concession	Eligibility	Interview	Diary	Interview	Diary	Interview	Diary
			*Frequencies*					

Intervention 1: Change from half-price to free travel:								

2002	Half-price travel (pre-intervention)	Age 60+ years^1^	2,355^2^	1,925	980^2^	825	1,571^2^	1,338
			
2003			2,344	2,037	977	884	1,661	1,482
			
2004			2,331^2^	2,056	1,003^2^	887	1,639^2^	1,474
			
2005			2,498	2,125	1,064	938	1,772	1,601

2006	Free travel (post-intervention)	Age 60+ years	2,500	2,143	1,119	978	1,662	1,495
			
2007			2,383	2,082	1,171	1,058	1,747	1,555
			
2008			2,271	1,989	1,211	1,086	1,765	1,582
			
2009			2,394	2,142	1,294	1,147	1,747	1,562

Intervention 2: Withdrawal of concession from 60−64-year-olds:								

2006	Free travel	Age 60+ years (pre-intervention)	2,500	2,143	1,119	978	1,662	1,495
			
2007			2,383	2,082	1,171	1,058	1,747	1,555
			
2008			2,271	1,989	1,211	1,086	1,765	1,582
			
2009			2,394	2,142	1,294	1,147	1,747	1,562

2010	Free travel	Rising eligibility age (post-intervention)	2,133	1,859	1,273	1,152	1,790	1,621
			
2011			2,243	1,989	1,177	1,051	1,703	1,522
			
2012			2,308	2,032	1,171	1,056	1,950	1,783
			
2013			2,129	1,882	1,113	1,013	1,954	1,793
			
2014			2,304	2,040	1,014	910	1,968	1,793
			
2015			2,457	2,058	1,073	892	2,088	1,810
			
2016			2,337	2,010	1,066	951	1,935	1,727

*Notes*: 1. In 2002 the eligibility age was 60 for women and 65 for men but this was equalised to 60 for both sexes in 2003. 2. Data on frequency of bus travel was not collected in 2002 and 2004.

**Table 2:** Characteristics of anaytical sample based on 50–74 year olds living in England and surveyed between 2002 and 2016

Interview data	50–59 year olds (Total N = 34,987)	60–64 year olds (Total N = 16,706)	65–74 year olds (Total N = 26,952)
Sex (%)			
Male	49.5	49.3	47.5
Female	50.5	50.7	52.5
Occupation (%)			
Manual/routine/none	40.7	44.2	47.7
Non manual	59.3	55.8	52.3
Rurality (%)			
Urban	79.6	77.4	77.6
Rural	20.4	22.6	22.4
Acces to car (%)			
Access	79.8	77.1	69.0
No access	20.2	22.9	31.0
Bus pass (%)			
No	100.0	49.1	25.9
Yes	0.0	50.9	74.1
Bus travel at least weekly (%)			
No	81.5	73.1	65.1
Yes	18.5	26.9	34.9
7-day diary data	50–59 year olds (Total N = 30,369)	60–64 year olds (Total N = 14,828)	65–74 year olds (Total N = 24,138)
Any bus or London underground travel^1^ (%)			
No	82.2	75.8	69.9
Yes	17.8	24.2	30.1
N journeys by car, van or motorcycle			
Mean (SD)	14.5 (10.2)	13.3 (9.7)	11.7 (9.1)

1 Reflecting additional benefits of London Freedom Pass
