# Supplementary material for: Impact of the statutory concessionary travel scheme on bus travel among older people: a natural experiment from England
Source: Ageing Soc. Author manuscript; Available in PMC 2020 Nov 1. (PMC7116195; doi:10.1017/S0144686X19000692)
Supplement: Supplementary [file EMS85068-supplement-Supplementary.docx]

 Supplementary Figure S1: Self-reported bus pass ownership according to survey year and age group, presented separately by sex, occupation, rurality and access to car

 Supplementary Figure S2: Self-reported weekly bus use according to year and age group, presented separately by sex, occupation, rurality and access to car

Supplementary Table S1: Change per year in frequency of bus pass ownership by period and age group

| Age group | Change per year in % owning bus pass | | Difference in change per year in % owning bus pass comparing post-intervention period with pre-intervention period in 60-64 and 65-74 versus 50-59 year olds |
| --- | --- | --- | --- |
|  | Pre-intervention | Post-intervention |  |
|  | | | |
| **Intervention 1: Change from half-price to free travel** | | | |
| 50-59  60-64  65-69 | 0.0 (0.0, 0.0)  3.3 (2.0, 4.7)  0.8 (-0.3, 1.8) | 0.0 (0.0, 0.0)  6.6 (5.3, 7.8)  5.1 (4.2, 6.1) | Reference  3.4 (1.7, 5.0)  4.5 (2.9, 6.1) |
| *p* |  |  | *<0.001* |
|  |  |  |  |
| **Intervention 2: Withdrawal of concession from 60-64 year olds** | | | |
| 50-59  60-64  65-69 | 0.0 (0.0, 0.0)  6.6 (5.3, 7.8)  5.1 (4.2, 6.1) | 0.0 (0.0, 0.0)  -7.5 (-8.0, -6.9)  -0.9 (-1.3, -0.6) | Reference  -14.1 (-15.1, -13.0)  -6.6 (-7.6, -5.5) |
| *p* |  |  | *<0.001* |
|  |  |  |  |

Supplementary Table S2: Change per year in frequency of weekly bus use by period and age group

| Age group | Change per year in % traveling weekly by bus | | Difference in change per year in % traveling weekly by bus comparing post-intervention period with pre-intervention period in 60-64 and 65-74 versus 50-59 year olds |
| --- | --- | --- | --- |
|  | Pre-intervention | Post-intervention |  |
|  | | | |
| **Intervention 1: Change from half-price to free travel** | | | |
| 50-59  60-64  65-69 | -0.4 (-1.5, 0.7)  -1.6 (-3.4, 0.3)  -0.3 (-1.8, 1.2) | 0.1 (-0.6, 0.8)  1.6 (0.5, 2.7)  2.2 (1.2, 3.2) | Reference  2.6 (0.0, 4.9)  1.2 (-0.1, 3.5) |
| *p* |  |  | *0.09* |
|  |  |  |  |
| **Intervention 2: Withdrawal of concession from 60-64 year olds** | | | |
| 50-59  60-64  65-69 | 0.1 (-0.6, 0.8)  1.6 (0.5, 2.7)  2.2 (1.2, 3.2) | -0.3 (-0.6, -0.0)  -1.8 (-2.2, -1.3)  -0.8 (-1.2, -0.4) | Reference  -2.9 (-4.1, -1.7)  -2.9 (-4.1, -1.7) |
| *p* |  |  | *<0.001* |
|  |  |  |  |

Supplementary Table S3a: Change per year in frequency of weekly bus/underground travel for any purpose by period and age group

| Age group | Change per year in % traveling weekly by bus/underground for any purpose | | Difference in change per year in % traveling weekly by bus/underground for any purpose comparing post-intervention period with pre-intervention period in 60-64 and 65-74 versus 50-59 year olds |
| --- | --- | --- | --- |
|  | Pre-intervention | Post-intervention |  |
|  | | | |
| **Intervention 1: Change from half-price to free travel** | | | |
| 50-59  60-64  65-69 | -0.3 (-1.1, 0.4)  -1.9 (-3.1, -0.6)  -0.7 (-1.7, 0.3) | 0.4 (-0.3, 1.2)  1.3 (0.1, 2.4)  1.3 (0.2, 2.3) | Reference  2.2 (0.4, 4.1)  0.7 (-1.1, 2.6) |
| *p* |  |  | *0.06* |
|  |  |  |  |
| **Intervention 2: Withdrawal of concession from 60-64 year olds** | | | |
| 50-59  60-64  65-69 | 0.4 (-0.3, 1.2)  1.3 (0.1, 2.4)  1.3 (0.2, 2.3) | -0.1 (-0.4, 0.2)  -1.5 (-2.0, -1.0)  -0.5 (-0.9, -0.1) | Reference  -2.2 (-3.4, -0.9)  -1.5 (-2.8, -0.2) |
| *p* |  |  | *0.003* |
|  |  |  |  |

Supplementary Table S3b: Change per year in frequency of weekly bus/underground travel for shopping or access to services by period and age group

| Age group | Change per year in % traveling weekly by bus/underground for shopping or access to services | | Difference in change per year in % traveling weekly by bus/underground for shopping or access to services comparing post-intervention period with pre-intervention period in 60-64 and 65-74 versus 50-59 year olds |
| --- | --- | --- | --- |
|  | Pre-intervention | Post-intervention |  |
|  | | | |
| **Intervention 1: Change from half-price to free travel** | | | |
| 50-59  60-64  65-69 | -0.1 (-0.6, 0.5)  -1.9 (-3.0, -0.9)  -0.3 (-1.2, 0.7) | -0.2 (-0.8, 0.3)  1.2 (0.2, 2.2)  1.1 (0.2, 2.0) | Reference  3.2 (1.7, 4.7)  1.0 (-0.5, 2.5) |
| *p* |  |  | *<0.001* |
|  |  |  |  |
| **Intervention 2: Withdrawal of concession from 60-64 year olds** | | | |
| 50-59  60-64  65-69 | -0.2 (-0.8, 0.3)  1.2 (0.2, 2.2)  1.1 (0.2, 2.0) | -0.3 (-0.5, -0.1)  -1.1 (-1.5, -0.7)  -0.6 (-0.9, -0.2) | Reference  -2.2 (-3.3, -1.2)  -1.7 (-2.7, -0.6) |
| *p* |  |  | *<0.001* |
|  |  |  |  |
